# Landfill site selection using a hybrid system of AHP-Fuzzy in GIS environment: A case study in Shiraz city, Iran

**DOI:** 10.1016/j.mex.2019.06.009

**Published:** 2019-06-14

**Authors:** Hasan Pasalari, Ramin Nabizadeh Nodehi, Amir Hossein Mahvi, Kamyar Yaghmaeian, Zabihalah Charrahi

**Affiliations:** a Research Center for Environmental Health Technology, Iran University of Medical Sciences, Tehran, Islamic Republic of Iran; b Department of Environmental Health Engineering, School of Public Health, Tehran University of Medical Sciences, Tehran, Islamic Republic of Iran; c Center for Solid Waste Research, Institute for Environmental Research, Tehran University of Medical Sciences, Tehran, Islamic Republic of Iran; d Department of Natural Resources, School of Environmental Sciences, Tehran University, Tehran, Islamic Republic of Iran

**Keywords:** Landfill site selection with AHP-Fuzzy in GIS, AHP-Fuzzy, Landfill site selection, GIS, Shiraz

## Abstract

Landfilling with simplicity and economic advantages is the most common element for waste management in both developed and developing countries. Landfill site selection in a proper way is an important municipal planning process which prevent environmental issues including water pollution imposed for insanitary landfills. The present research was developed to exhibit a simplified method of multi criteria decision making (MCDM) and Fuzzy memberships in GIS environment to ascertain best landfill sites for Shiraz county, located south of Iran. 15 most common sub-criteria, documented in literature and implicated by Iranian environmental protection organization (IEPO) including surface water, ground water, land use, distance to well, soil type, slope, protected area, fault in environmental group, residential area, road, airport, village, infrastructure, historical area, wind direction in socio-economical group were selected and the weight of each criterion was determined based on expert’s knowledge with use of analytical hierarchy process (AHP). The results of the present research are as follows:

• Distance to residential area and groundwaters with weight of 0.36 and 0.28 were recognized as the most important criteria for landfill site selection.
• The six suitable areas for landfill in Shiraz county is 1.003% of total area equal to 8710 ha.
• AHP and Fuzzy memberships has a great potential and ability for landfill site selection.

## Highlights

• The utility of novel technologies seems imperative to select a suitable site for landfill as new criteria are increased.
• Distance to residential area and groundwaters with weight of 0.36 and 0.28 were recognized as the most important criteria for landfill site selection.
• The six suitable areas for landfill in Shiraz county is 1.003% of total area equal to 8710 ha.

## Graphical abstract

**Figure:**
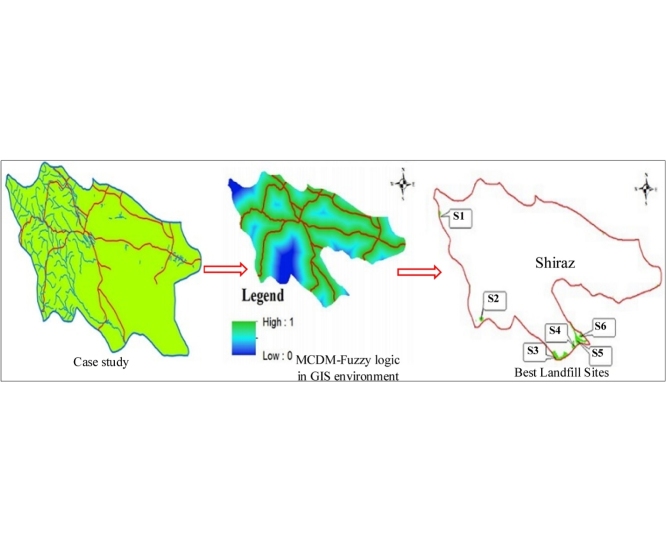

**Specifications Table**

**Table:** 

Subject area:	Environmental sciences (Solid waste management)
More specific subject area:	Landfill site selection
Method name:	Landfill site selection with AHP-Fuzzy in GIS
Name and reference of the original method:	Z.K. Motlagh, M.H. Sayadi, Siting MSW landfills using MCE methodology in GIS environment (Case study: Birjand plain, Iran), Waste Manag. 46 (2015) 322–337. doi:10.1016/j.wasman.2015.08.013.
Resource availability:	Data is presented in this article

## Method details

In most developing countries such as Iran, waste disposal is still being applied for waste management due to lack of enough knowledge and equipment to manage waste. However, this method, if applied inappropriately, could cause some problems to both human health and the environment, which are in opposite to sustainable development goals (SDGs) [1]. Waste generation, storage and disposal are major causes of spreading diseases and pollution of ground-and-surface water resources [2]. Although recycling and reuse, as two main elements of waste management, are extensively applied in most developed countries, authorities in developing counties, in particular Iran have no alternatives to choose and must select the landfill site for waste management. On the top of this, existing much land around the city necessitate the authorities to take advantages of this element, landfill site [3,4]. Iran has a lot of land which can be applied for waste disposal, however, the population is growing and the cities are increasingly growing as well [5]; for this reason, a method by which the experts can find the best suitable place for landfill is of great importance in waste management system [6,7]. In addition, Shiraz has many world-heritages such as *Takht-E-Jamshid*, *Perspolis* complex place and etc., and the authorities are required to employ the high-accuracy method in order to select the best probable site for landfill [8].

The use of novel technologies to reduce the adverse effects arising from municipal solid waste in Iran and other developing countries seems to be necessary and imperative. This task can be more convenient and less time-consuming by using integration of knowledge of local experts involving in waste management practices and multi-criteria decision analysis (MCDA) methods [[9], [10], [11], [12]]. Many research have focused on some kinds of fuzzy-logic abilities to find the most likely suitable area for landfill to improve the knowledge of decision-making process [[13], [14], [15], [16]]. However, these research provide the landfill site selection in a complicated way, which is not kind of user-friendly. The present study aimed at developing a site selection method based on a GIS- AHP-Fuzzy hybrid. For this purpose, the basic rules were extracted from Iranian environmental protection organization (IEPO) and integrated with the knowledge of local experts to simplify the landfill site selection process, of note, these criteria are in consistent with EPA criteria and most-applied criteria in the manuscripts published [17]. A questionnaire was designed to extract the experts' knowledge and also obtain weighting factors for each parameter using AHP method. The method was applied to cover the environmental and socio-economical criteria in order to simplify a high accuracy landfill site selection method in GIS environment and to be user-friendly for authorities involved in waste management in developing countries including Iran and the Shiraz County.

## Background information

**Fig. 1:**
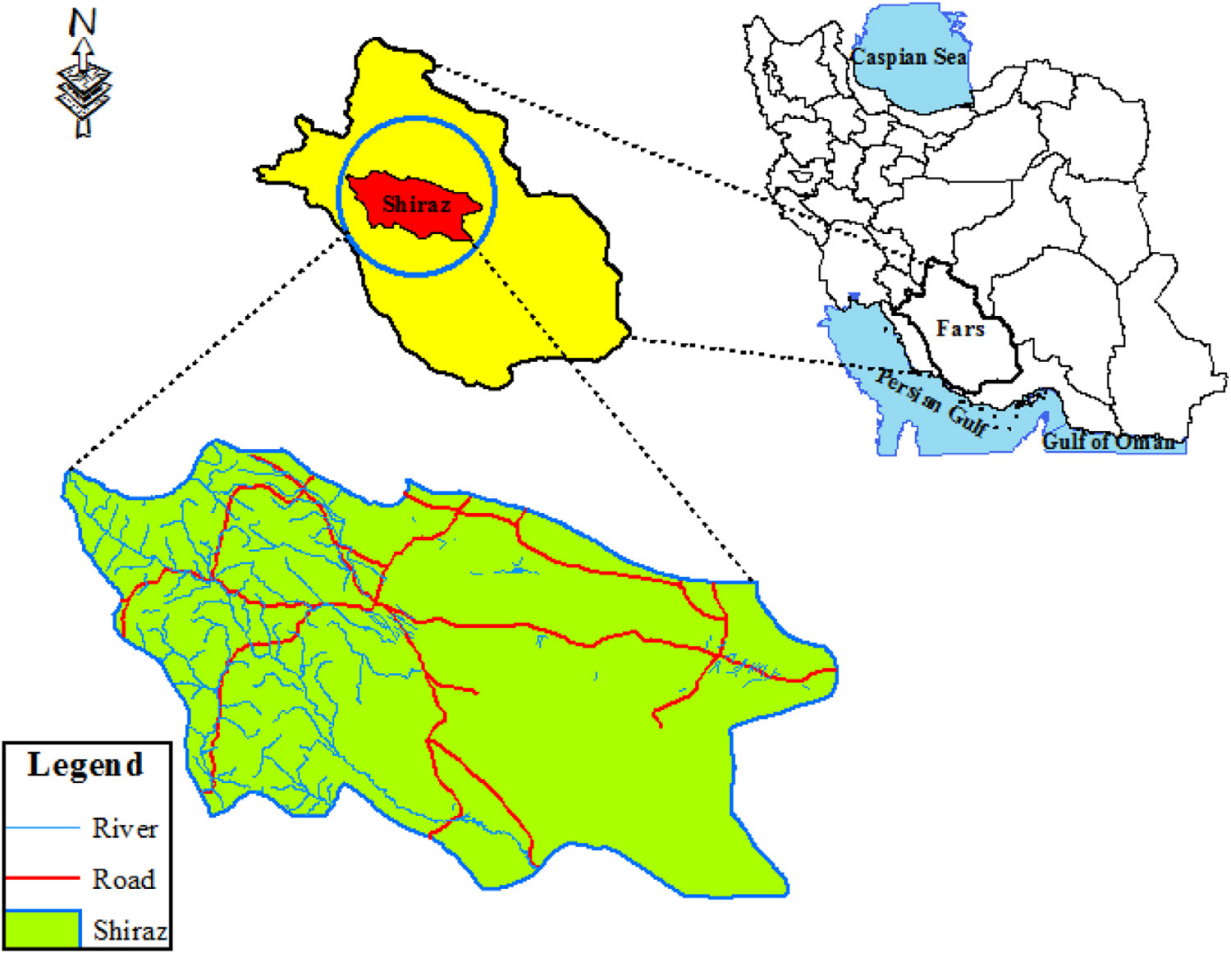
The local map of the study area, Shiraz.

Shiraz county is located on center of Fars province, Iran in geographical coordinates, 51. 791°–53. 593 °E longitude and 29.446°–33.100 °N latitude as seen in Fig. 1. General information and characteristics including demographic and meteorological on case study are described in Fig. 1. The waste produced daily in Shiraz, as sixth most populous city of Iran, with current population is over 954 tons.

The northeast (NE) and southwest. (SW) winds are the most common wind directions in Shiraz city. In terms of cultural and tourism perspective, Shiraz area is known as a city with some cultural heritages of global importance such as *Takht-jamshid, Perspolis* complex palaces which attract over a million tourists all over the world throughout a year. A recent solid waste qualitative analysis in Shiraz indicated that of 954 tons waste generated daily, a highly percentage equal to 70.63% (by dry-weight) was organic matters.

Currently, there are no waste-source separation, reuse, recycling techniques to manage the waste. In addition, due to large area around the city, authorities have no option and alternative to utilize; they are made to dispose of waste in sanitary landfill. The only landfill site of Shiraz used currently for waste management is placed in 52° 53′ E longitude 29°52′ N latitude. Due to environmental hazards including lack of technologies to manage leachate and landfill gases causing detrimental impacts on locals and global community, it is expected to displace current landfill to another suitable place. Furthermore, Shiraz similar to most cities in developing countries, the municipalities faces problem such as budget capacity to manage the waste generated. Therefore, they are week in economic perspective to handle the heavy expenses associated with landfill site.

## Siting methodology

Considering limitations descripted by Iranian environmental protection organization (IEPO) and the knowledge of local experts, the construction decision making tree was developed. These limitations will be descripted separately in any section related to corresponding criterion.

### Data collection, developing a decision making tree and criteria weighting

**Fig. 2:**
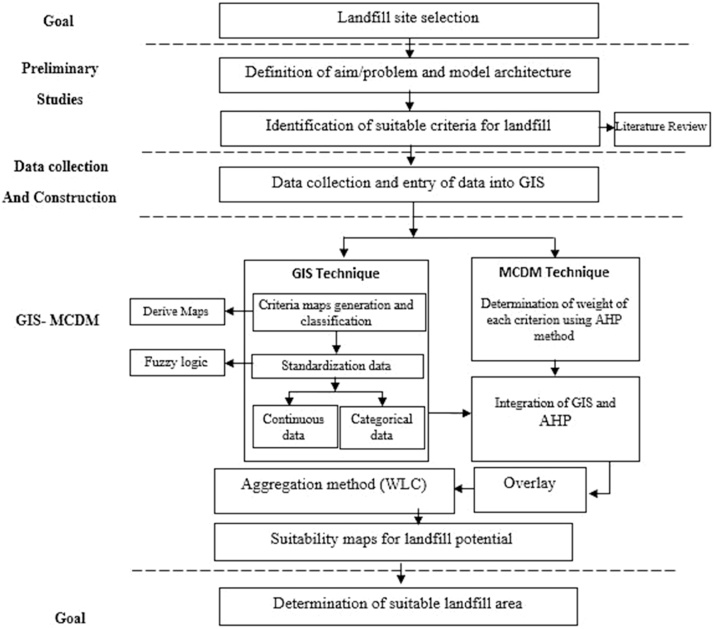
The hierarchical structure used in the study area for modeling suitable landfill site selection in GIS environment.

In this present study, 15 most common and applicable criteria, documented in the published literature, consistent with IEPO criteria and expert knowledge familiar to case study situations were selected for this area study and schematized within two groups. These groups are called environmental and socio-economical criteria. The conceptually hierarchical scheme model of this site selection is illustrated in Fig. 2.

Then, a questionnaire containing information about the role of each criterion in landfill site selection was designed. The questionnaire results of 20 experts involved in waste management and familiar with local situations from various organizations, including IEPO, municipalities, stakeholders, environmental health engineering professors and students were entered to expert choice program and the weight of each criterion and sub-criterion were determined, as shown in Table1.

**Table 1:** Factors and their weights obtained from AHP analysis for landﬁll site selection.

Criteria	weight	Sub-criteria	weight	Data sources	References
Environmental	0.75	Surface water	0.23	Fars Water organization	[20,21]
		Ground water	0.28	Fars Water Organization	[19,22]
		Land use	0.09	Ministry of Interior	[18,23]
		Distance to Well	0.17	Fars Water organization	[6,24]
		Soil type	0.06	Iran Water Organization	[25,26]
		Slope	0.02	Iran Water Organization	[27,28]
		Protected area	0.07	National Cartographic Center	[22,29]
		Fault	0.04	Iran Water Organization	[30]
Sum			**1.00**		
Consistency Rate			**0.09**		
socio-economical	0.25	Residential area	0.36	Ministry of Interior	[19,31]
		Road	0.20	National Cartographic Center	[32]
		Airport	0.05	National Cartographic Center	[33,34]
		Village	0.16	Ministry of Interior	[35]
		Infrastructure	0.03	National Cartographic Center	[18]
		Historical area	0.07	National Cartographic Center	[6,36]
		Wind direction	0.13	Fars Meteorological Organization	[22,37]
Sum	**1.00**		**1.00**		
Consistency Rate			**0.08**		

In the present research, analytical hierarchy process (AHP) method was employed to calculate the weight of environmental and socio- economical criteria. AHP is based on pairwise comparison and widely accepted and used in decision making for different aims. AHP introduced by *Saaty (1980)* [18], was one of the useful and applicable methodologies and can play a considerable role in selecting alternatives.

This method was employed to ascertain the relative importance of each sub-criterion in selection the best probable sites for landfill. AHP uses an index named consistency ratio (CR) to represent the overall consistency of the pairwise comparison matrix. The CR with a value of less than 10% are acceptable to emphasize the consistence method [19]. As viewing of Table 1, this indicator for the present study is lower than the value considered for consistency of method.

Respective maps to selected criteria were taken from different related organizations and digitalized. These maps include surface water, ground water table, land use, well, soil type, slope, protected area, fault, residential area, road, airport, village, infrastructure, historical area, wind direction, which are summarized in Table 1.

### Application of GIS in landfill candidate site selection (Entry of data into GIS)

While landfill site selection with different methods has been extensively investigated worldwide, but no internationally accepted integrated approach is available to be applicable for waste disposal in every country's conditions. In the present research, a GIS database was created for landfill site selection via AHP-Fuzzy in GIS (Version 10.3) environment after obtaining criteria from various sources, and then prepared as map layers containing format projection system of UTM-39 N. For the preparation, reclassification and performing many functions in accordance with IEPO regulations on the needed layers, these maps should be converted to raster-based ones.

### Developing GIS-AHP-Fuzzy hybrid model of site selection

As the date used in this study were gathered from different sources with different formats, in the first step of MCDA, we standardized all database in a comparable format. To make attribute layers of criteria comparable, we can use a variety of approaches [38]. In the present research, according to previous studies and literatures relevant and experience of experts, fuzzy concept has been applied to standardize the criteria of data. Due to flexibility in concept of Fuzzy logic, it is suitable for data modeling [39] and users can define the element with no limitation in assigning values which are typically between 0 and 1 [40]. In such cases, the elements belonging to the target criteria are defined on the basis of the degree of membership of a particular function (Small, Large, Gaussian, Linear or user-defined). In addition, the selected membership functions will be compatible with the nature, limitations defined for criteria and thresholds of membership functions. Linear or sigmoidal functions are broadly selected and sufficient in the process of decision making particularly for landfill site selection strategy. In this study, for criteria with categorical values including geology cover, soil cover, land use, a distinct classification has been applied, so that the values of the elements of fuzzy set were selected based on experts’ knowledge. For all other criteria, whose element values gradually change from one location to another, a fuzzy membership function through a linear transformation was applied to classify; these values are user-specified and defined between the minimum value as a membership of 0 and maximum value as membership of 1. Selecting point markers properly is a need to define the degree of membership. There are often four point markers in the fuzzification and membership function: the first point mark (a) is location where the membership function starts to rise above 0. The second point mark (b) demonstrates the place where it is approaching to 1. The third point mark (c) shows the location where the membership grade begins to drop again below 1, while the fourth point mark (d) indicates where it returns to 0, as shown on Table 2.

**Table 2:** Summary of Fuzzy standardization for criteria.

Cluster	Criteria	Fuzzy and shape membership functions	Control point/ Value point
Environmental	Surface water	Increasing – Linear	a = 1000 m
			b = 20,000 m
	Ground water	Increasing – Linear	a = 10 m
			b = 300 m
	Land use	user defined	-----
	Distance to Well	Increasing – Linear	a = 40 m
			b = 3000 m
	Landform	user defined	------
	Slope	Reducing - Linear	a = 68°
			b = 20°
	Protected area	Increasing – Linear	a = 1000
			b = 6000
	Fault	Increasing – Linear	b = 40,000
Socio-economical	Residential area	Increasing – Linear	a = 1000
			b = 50,000
	Road	Reducing – J-Shape	a = 1000
			b = 3000
			c = 25,000
	Airport	Increasing – Linear	a = 8000
			b = 10,000
	Village	Increasing – Linear	a = 1000
			b = 50,000
	Infrastructure	Increasing – Linear	a = 500
			b = 80,000
	Historical area	Increasing – Linear	a = 3000
			b = 50,000
	Wind direction	user defined	-------

#### Environmental criteria

##### Surface water

**Fig. 3:**
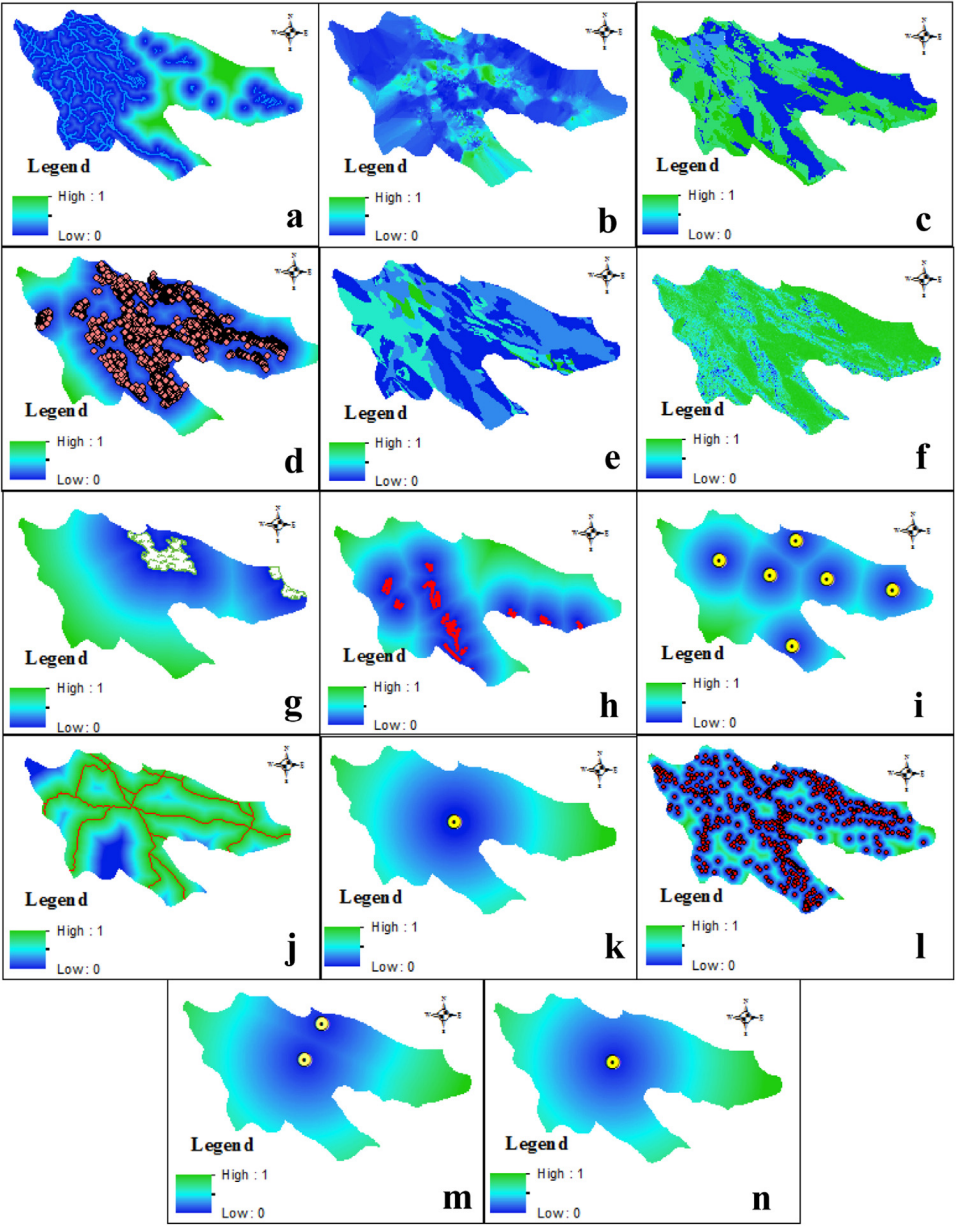
Membership value trend assigned to criteria: (a) Surface water, (b) Ground water, (c) Landuse, (d) all wells, (e) Landform, (f) Slope, (g) Protected area, (h) Fault, (i) Urban area, (j) Road, (j) Airport, (k) Village, (l) Infrastructure, (m) Infrastructure, (n) Historical area.

Landfills can significantly threat surface-water sources, if inappropriately designed [41]. The waste containing chemicals and byproducts that are made from existing reactions in the landfill can be dissolved in water and consequently pollute the discharged leachate [42]. Leachate are able to emerge in water and make it contaminated [43,44]. So, people consuming and are in contact with this contaminated water are subject to a risk which endanger them. Ecological resources are extremely affected by the contaminants present in uncontrolled landfill leachate. Surface water resources contaminated with pollution arising from waste disposal are often with low dissolved oxygen level. Thus, it can make the situations suitable for attraction of disease-carrying organism and consequently lower the ecological health of water bodies [45]. IEPO regulates that water sources must be 1000 m far away the selected landfill. Euclidean distance was applied to determine the continues distances away from or outward distance from surface waters. To standardize distances from surface water like rivers, lakes and springs, an increasing- Linear fuzzy function was employed, as represented in Table 2. The first control point (a = 1000 m) indicates the least suitable distance for siting a landfill while the second control point (b = 20,000 m) as the farthest distance from water sources indicates the most suitable distance for siting a landfill. Fig. 3(a) shows the membership value trend calculated for categories considered for analysis of surface water bodies.

##### Ground water

A system with capability of regular monitoring the groundwater surrounding landfill is necessary to ensure whether the lined system considered for collecting leachate works properly [45]. Many factors can make the groundwater polluted and lower the quality, ultimately is not suitable for special activities and drinking [46,47]. The transportation of landfill pollution following different reactions occurred into the groundwater and environment are main concerns of interest in developing countries, which need measurement to meet the criteria required for safe environment. In this work, we used a total 10 m depth from ground to protect the groundwater from pollution. Fig. 3(b) shows the membership value trend calculated for categories considered for analysis of ground water.

##### Landuse

Landuse can be used as resolving agent to satisfy people to accept the unwanted facility. Actually, the land use with low value in public perspective creates less resistance to place for landfill [48]. As IEPO regulations about landfill site selection, landfill must not be selected in some land uses, including agricultural and forest. The land use in this study area was developed with a fuzzy method which were given scores between 0–256, as the importance of criterion in landfill site selection. Barren landuse dominates the Shiraz area. As listed in Table 3, the high value was assigned for barren landuse as most suitable landuse while other landuses are of importance in the area. Table 3 shows the values considered for categories of landuses for analysis.

**Table 3:** User-assigned values to land use importance for landfill site selection.

Land use	Intermediate Grassland	Half-density forest	Barren Land	Salt Marsh	Scattered trees
Value	70	180	190	220	240

The data were standardized and normalized with Eq. (1). With this equation, various land uses take value between 0 and 1. Normalized land uses were converted to raster with Feature to Raster technique.

Fig. 3(c) shows the membership value trend calculated for categories considered for analysis of landuse.

(1) $$\frac{X_{i}-X_{\min}}{X_{\max}-X_{\min}}$$

Where, $X_{i}$ is the value of variation, $X_{\min}$ and $X_{\max}$ are respectively the lowest and highest values of value importance of variation.

##### Distance to well

Wells are extremely important to prepare the water sources for activities and drinking water, and they are influenced by many factors including, agricultural [49] and reactions occurred in landfill sites. Landfill must be 40 m away from the wells to prevent the probable contaminations. Distance between 0–40 m is specified with 0 value and the farther distance has greater value to 1. Fig. 3(d) shows the membership value trend calculated for categories considered for analysis of distance to wells. There are over 10,000 wells over the Shiraz county.

##### Landform

The landform map was digitized using overlay of geology map and satellite map in ArcGIS environment and converted into a grid map with a 30_30 m resolution. Although there are different formations in Shiraz area, they are classified in eight landform units, namely, lagoon & salt bottom, Karstic limestone, Salt dome, Alluvium terrace, Medial plain, Sandstone, Plain with hill, and bedrock with low permeability. Alluvium and karstic limestone landform due to high potential to water adsorption are often not considered for landfill sites. suitable for landfill sites. Table 4 shows the values considered for categories of landform for analysis.

**Table 4:** User-assigned values to landform importance for landfill site selection.

Landform	Lagoon & salt bottom	Karstic limestone	Salt dome	Alluvium terrace	Medial plain	Sand stone	Plain with hill	Stony & bedrock with low permeability
Value	10	10	110	50	50	80	100	230

The data were standardized and normalized with Eq. (2). With this equation, various landuses take value between 0 and 1. Normalized land uses were converted to raster with Feature to Raster function in GIS environment. Fig. 3(e) shows the membership value trend calculated for categories considered for analysis of landforms.

(2) $$\frac{X_{i}-X_{\min}}{X_{\max}-X_{\min}}$$

Where, $X_{i}$ is the value of variation, $X_{\min}$ and $X_{\max}$ are respectively the lowest and highest values of value importance of variation.

##### Slope

The slope is considered as an important criterion according to IEPO regulations to select the best site for landfill, especially for landfill construction and operation purposes. In the present study the slope degree for Shiraz area was derived from preprocessed ASTER G-DEM (asterweb.jpl.nasa.gov) [50]. In this study area, the high values were assigned to area with slope less than 20% and as the slope become more, the value decreased. Shiraz is a town with slope between 0 and 68 degrees. Fig. 3(f) shows the membership value trend calculated for categories considered for analysis slope criterion.

The slope was standardized by reducing- Linear fuzzy function controlled by two points (a = 20%, b = 68%) where slops less than 20% are the most suitable (full membership) and more than 20% are not suitable (full-non-membership).

##### Protected area

Accordance to IEPO, national parks and historical area must not be applied as landfill. Landfill must be at least 1000 m far away these sites. For this criterion, we considered value 0 for distance less than 1000 m and as distance increased the value became near to 1, the highest value.

Fig. 3(g) shows the membership value trend calculated for categories considered for analysis of protected area.

##### Fault

Distance to faults in events such as earthquake has an important role in preventing spreading pollution and deconstruction of site building.

Euclidean distance was applied to determine the continues distances away from or outward distance from fault lines. To standardize distances from fault lines, an increasing- Linear fuzzy function was employed, as represented in Table 1. The high values were assigned to the farthest distance from fault and obtained the 1 value. Fig. 3(h) shows the membership value trend calculated for categories considered for analysis of Fault lines.

#### Socio-economical criteria

##### Residential area (Urban area)

selecting a landfill near a residential and urban area can cause a variety of environmental unwanted odor and noise pollution [39]. As criteria in landfill site selection should be in line with IEPO requirements to landfill site, residential area must be buffered at least 1000 m away from landfill site. Fig. 3(i) shows the membership value trend calculated for categories considered for analysis of residential area.

##### Road

Landfill sites must be in access in any weather conditions. The further away landfill is placed from the road and accessibility to landfill is difficult, it can consequently rise the cost associated with transportation and is not of interest for authorities involved in waste management. However, landfills closer to road make an aesthetically bad view.

Euclidean distance was applied to determine the continues distances away from or outward distance from existing roads. The higher membership values were assigned to closer distance and inversely with lower ones were considered for more away distant places. A j-shaped decreasing function was used where distances of less than 1000 m were assigned a value of 0 while for distances exactly 1000 m, the value membership was considered 1 and the distances beyond 1000 m and more the membership values begin to drop below with approaching trend to zero **(**Table 1). Fig. 3(j) shows the membership value trend calculated for categories considered for analysis of distance from roads.

##### Airport

The distance between an airport and landfill must be more than 8 km. Fig. 3(k) shows the membership value trend calculated for categories considered for analysis of distance from airport. Distances with less than 8000 m has less values and with increases in distances this value increases close to 1.

##### Village

IEPO recommend that the landfill for disposal of waste is better to be located at least 1000 m away from the residential area and villages. As a criterion in landfill site selection should be in consistent to IEPO requirements to landfill site, residential area must be buffered at least 1000 m from landfill. Euclidean distance was applied to determine the continues distances away from or outward distance from villages. To standardize distances from the villages, an increasing- Linear fuzzy function was employed, where distances of less than 1000 m from the edge of the urban areas were assigned a value of 0. Fig. 3(l) shows the membership value trend calculated for categories considered for analysis of distance from the villages.

##### Infrastructure

Landfill sites must be placed away from industrial and military area. In this study distance more than 500 m was considered. Fig. 3(m) shows the membership value trend calculated for categories considered for analysis of distance from the infrastructures.

##### Historical area

As you know, Shiraz is the cultural capital of Iran and has many historical areas. In regard to IEPO's regulation, considering distance at least 1000 m between historical area and landfill is imperative. Fig. 3(n) shows the membership value trend calculated for categories considered for analysis of distance from the historical area.

##### Wind direction

The wind direction is of most important criteria in landfill site selection. The odor originating from a landfill can bother the residents living in the direction of wind. According to reports obtained from the National Meteorological Agency of Fars, the northeast (NE) to southwest (SW) winds are the dominant winds in the basin. Wind direction is the most commonly criterion used in site selection. In this research, wind direction criterion was selected based on IEPO criteria, in consistent with literatures documented and compared with other criteria.

## Results

**Fig. 4:**
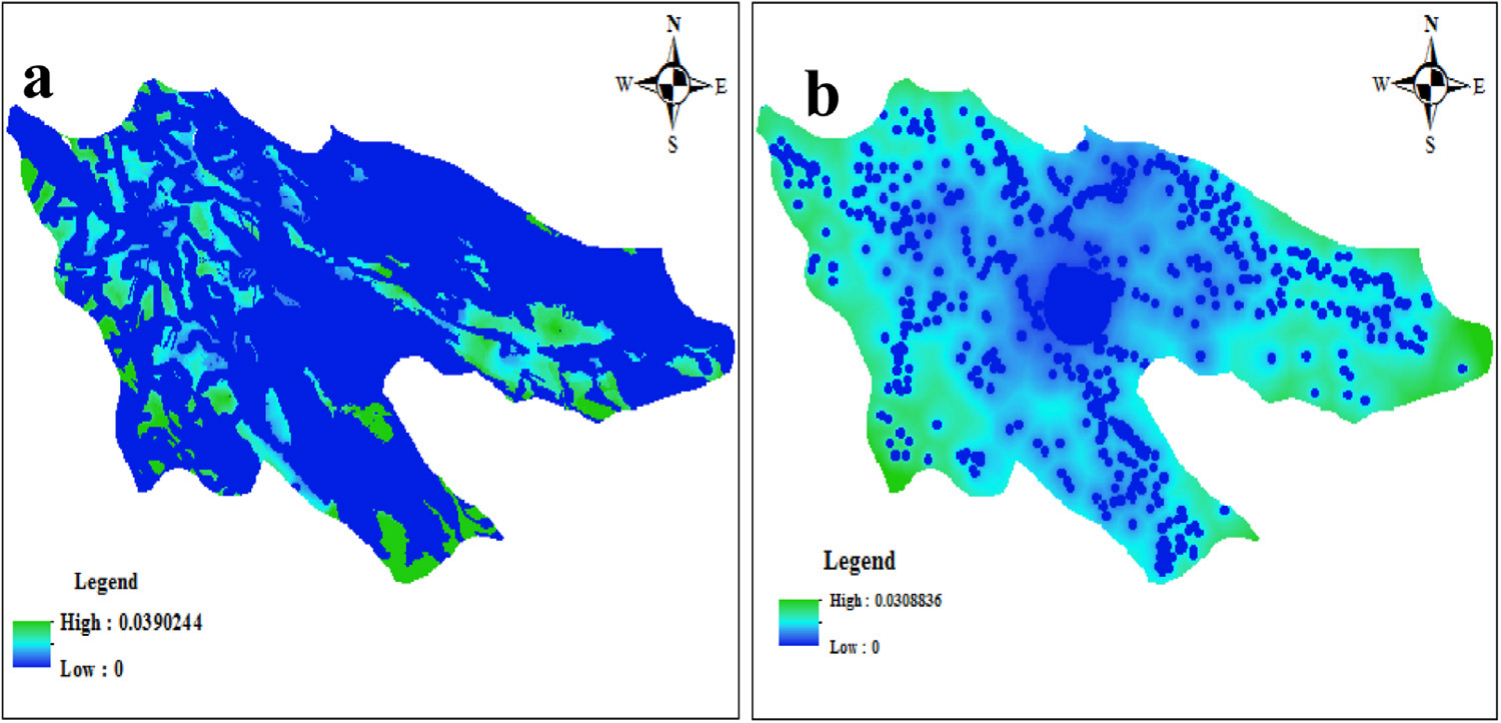
(a) and (b): obtained overlay maps based on environmental (a) and socio-economical (b) constrains.

In this current research, a variety of environmental and socio- economical criteria were used for the landﬁll site selection, addressed in the Table 1. The obtained overlay map based on environmental and socio-economical constrains addressed in Table 1 are depicted in Fig. 4(a) and (b), respectively. The weights of criteria and sub-criteria in both environmental and socio-economical are based on weight obtained from the AHP method. Overlaying maps were conducted with various Gamma functions in Arc-GIS. However, we achieved the best results with Gamma function 0.8. By looking at this map, it can be realized that most of the study area does not have the suitable properties which were considered in different criteria for landfill construction.

Accordingly, only a limit area of the study area can be evaluated in more details based on the factors, which cover approximately 1.003% of total area equal to 8710 ha of case study area. The suitable area for landfill in Shiraz county is a little less than that (2.67%) in Marvdasht city, located beside the case study [8]. The reason for this issue can be due to more existence of flat area in Marvdasht. Finally, the both resulting maps, environmental and socio-economical, were intersected with function overlay with each own weigh, which are 0.75 and 0.25 as idea of experts and the best sites for landfill site selection in Shiraz county were selected.

**Fig. 5:**
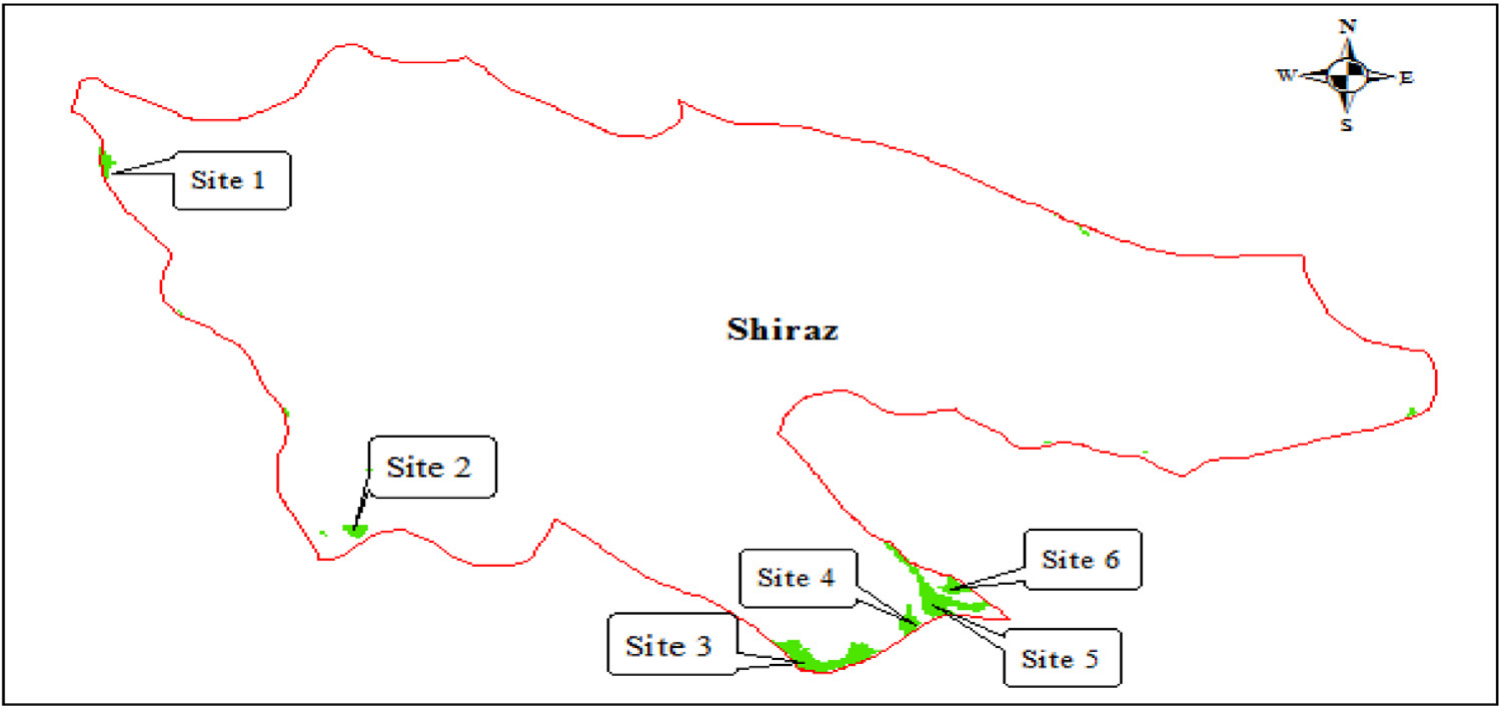
Best sites for landfill site based on intersection of associated maps to environmental and socio-economical criteria.

As shown in the Fig. 5. The six best probable sites were chosen for landfill sites. Considering the prevalent wind detection and required area for landfill for a 20-year design period, equal to 430 ha, these sites mostly placed in the south-eastern of shiraz county can be suggested to landfill in the future.

## Conclusions

At the end of the analyses, appropriate MSW landfill sites were identified. These sites were selected based on requirements enacted by IEPO for landfill selection in Iran. Among these six candidate sites, the best site can be selected with in depth investigation and field observation. The selection of the final MSW site, however, requires further geotechnical and hydrogeological analyses towards the protection of groundwater as well as surface water.

The results of present research indicated that the methodology applied is an effective and simple approach for landfill selection. In addition, the described methodology is user friendly and can employed by authorities in developing countries to lower both time and cost.
